# Clinical features and Surgical Outcome of Clear Cell Papillary Renal Cell Tumor: result from a prospective cohort

**DOI:** 10.1186/s12894-023-01216-7

**Published:** 2023-03-21

**Authors:** Si Hyun Kim, Jang Hee Han, Seung-hwan Jeong, Hyeong Dong Yuk, Ja Hyeon Ku, Cheol Kwak, Hyeon Hoe Kim, Kyung Chul Moon, Chang Wook Jeong

**Affiliations:** 1 Department of Urology, Seoul National University Hospital, Seoul, Korea; 2 Department of Urology, Seoul National University College of medicine, Seoul, Korea; 3 Department of Pathology, Seoul National University Hospital, Seoul national University College of medicine, Seoul, Korea; 4 Department of Urology, Seoul National University Hospital, Seoul national University College of medicine, 101 Daehak-ro, Joungno-gu, Seoul, 03080 Korea; 5 Department of Urology, Soonchunhyang University Cheonan Hospital, Cheonan, Korea

**Keywords:** Small renal mass, Renal cell carcinoma, Nephrectomy, Pathology, Surgical

## Abstract

**Background:**

Clear cell papillary renal cell tumor (CCPRCT) was first reported in 2006 a patient with end stage renal disease. After that it was discovered in the kidney without end stage renal disease in the 2010s and started to be mentioned in pathology and urology. The incidence of CCPRCT is low and most of it is discovered incidentally, so there is a lack of reports on clinical characteristics and surgical outcome.

**Methods:**

This study used clinical data from the Seoul National University Prospectively Enrolled Registry for Renal Cell Carcinoma-Nephrectomy (SUPER-RCC-Nx). Between August 2016 and July 2022, patients who underwent radical or partial nephrectomy with clear cell papillary RCC with pathological finding were included in this study. All patients’ pathologic reports were reviewed by 1 pathologist. Clinical characteristics and surgical outcomes were presented through descriptive statistics, and Kaplan-Meier curve used for survival analysis.

**Results:**

Of the 2057 patients, CCPRCT was reported in 36 patients (1.8%). The median follow up period was 26.8 months. The median age was 67 years, and there were 10 females and 26 males. The median tumor size was 1.2 cm. Twenty-nine patients underwent partial nephrectomy. Seven patients with end-stage renal disease underwent radical nephrectomy. The median operative time for patients who underwent partial nephrectomy was 97.5 min and the estimated blood loss was 100 cc. The median hospital days was 4 and 30-day complications were 2 cases with clavien-dindo classification III or higher. During the follow-up period, there was no recurrence and cancer specific mortality.

**Conclusions:**

The size of CCPRCT was small and there was no advanced stage at that time of diagnosis. There was no recurrence or cancer specific mortality during the follow-up period. A multi-center study with a large scale is needed in the future.

**Trial registration:**

Seoul National University Hospital (SNUH) Institutional Review Board (IRB) (approval number: 2210-126-1371).

## Background

Kidney cancer is the 8th most diagnosed tumor in the United States [1], and the incidence rate is steadily increasing in Korea, which is similar to that in the West [2]. Kidney cancer is a cancer that has no specific symptoms in early stage, and is likely to be very advanced when symptoms appear. Although the 5-year survival rate is reported to be about 71%, the number of patients who can be diagnosed and operated early with advanced imaging tests is increasing [3].

Kidney cancer, not only the stage but also histological classification greatly influences the prognosis. In 1952 there were two histological classifications of kidney cancer, but now there are more than 20 classifications [4]. Due to the rapid change in the classification system, many histological studies on newly classified renal cancer are being conducted, but information on clinical characteristics and follow-up is insufficient [5].

The World Health Organization (WHO) started using the renal epithelial tumor classification system in 2004, and in late 2006 began to classify it as clear cell papillary renal cell tumor (CCPRCT) in patients with end stage renal disease (ESRD) [4]. Since then, it has been reported in normal kidneys and is known to account for 1 to 4% of all kidney cancers. There have been no reports of metastasis so far, and the prognosis is expected to be very good, but studies are lacking [6]. It has good borders and is mainly surrounded by film. Microscopic findings show various tubular and papillary structures and the cytoplasm is empty. The Fuhrman nuclear grade is low, and the nuclei are arranged in rows away from the basement membrane. It can be distinguished from clear cell renal cell carcinoma (ccRCC) on Hematoxylin and Eosin slides, but immunohistochemistry is helpful. Mostly cytokeratin 7 (CK7) is expressed and alpha-methylacylCoA racemase (AMACR) and CD10 are negative [7].

CCPRCT was often found in real world. However, clinical information about it was limited [8]. This pathology is unfamiliar to some urology clinicians and the understanding of these pathological consequences is poor. The purpose of this study is to provide clinical, surgical and oncological information by investigating the characteristics of patients with these pathology outcomes based on the renal cell carcinoma surgery data of Seoul National University. It is hoped that this study will improve the understanding of CCPRCT and help determine future treatment directions.

## Methods

### Ethics approval and informed consent

This study was approved by the Seoul National University Hospital (SNUH) Institutional Review Board (IRB) (approval number: 2210-126-1371). The prospectively collected cohort was approved by the SNUH IRB (approval number: 1506-122-682) for use of clinical data for scientific purposes. Informed consent for this study was obtained from each participant. All experiments were performed in accordance with relevant guidelines and regulations.

### Study design and patients’ clinical data

This study used data from the prospective, multidisciplinary, and biobank linked cohort, the SUPER-RCC-Nx [9]. Between August 2016 and July 2022, patients who underwent radical or partial nephrectomy were included in this study. CCPRCT Patients were screened based on pathologic reports. The reports of the included patients were reviewed by 1 experienced pathologist.

The following clinical data were assessed: age, sex, body mass index, past medical history, family history, smoking history, end stage renal disease, Von Hippel Lindau (VHL) syndrome, computed tomography (CT) image, tumor location, size, number, R.E.N.A.L nephrometry Score [10], clinical stage, previous other cancer diagnosis history, follow-up period, perioperative outcomes, pathologic results, and complications.

### Immunohistochemistry

The immunohistochemical staining were performed with the following markers: CK7 (1:300, Dako, Glostrup, Denmark), CD10 (ready-to-use, Novocastra, Newcastle, UK), AMACR (1:300, Dako), transcription factor E3 (TFE3; 1:1,500, Santa Cruz Biotechnology Inc., Santa Cruz, CA, USA) and Vimentin (VT; 1:500, Dako). Each slide was dewaxed and rehydrated in a graded series of alcohol solutions. CK7, CD10, TFE3 and VT were subjected to immunohistochemical staining using Bond-Max autostainer (Leica Microsystems, Bannockburn, IL, USA). AMACR staining was performed using Dako Autostainer Link 48 (Dako Corp., Carpintera, CA, USA).

### Statistical analysis

Baseline characteristics were shown as descriptive statistics. From the time of diagnosis to the last follow-up, recurrence and survival rates were investigated and analyzed using the Kaplan Meier curve. All statistical analyses were performed using R software version 3.6.3 (R Foundation for Statistical Computing).

## Results

A total of 2057 patients underwent partial or radical nephrectomy during the study period, CCPRCT was reported in 36 patients (1.8%). The median follow up period was 26.8 (interquartile range (IQR): 12.7–49.5) months. The characteristics of CCPRCT patients are as follows. The median age was 67 (IQR: 56.5–71) years, and there were 10 females (27.8%) and 26 males (72.2%). Thirty-two patients (88.8%) presented to the hospital with an incidentally discovered renal mass. These patients had no specific symptoms. The masses were found incidentally during a health checkup or CT scan conducted by other departments. Among the patients, there were 7 patients (19.4%) with ESRD. The Eastern Cooperative Oncology Group performance status of all patients was 0 (100%). Eighteen patients (50%) had a history of smoking or were currently smoking.

VHL syndrome was found 1 patient (2.8%). Synchronous ipsilateral multiple masses were observed in 3 patients (8.3%). Synchronous bilateral masses were observed in 1 patient (2.8%). Metachronous CCPRCT was found in 1 patient (2.8%). In 4 patients (11.1%), CCPRCT was observed concurrently with ccRCC or another type of RCC, and 1 patient (2.8%) was found simultaneously with ccRCC. Patient characteristics are shown in Table 1.

**Table 1:** Basic characteristics of patients *IQR: Interquatile range; BMI: Body mass index; CCI: Charlson Comorbidity index; ECOG PS: Eastern Cooperative Oncology Group Performance status;VHL: Von Hippel Lindau; CCPRCT: clear cell papillary renal cell tumor; ccRCC: clear cell renal cell carcinoma

	Per patients (N = 36)	
Median Age, years (IQR)	67 (56.5–71)	
Median BMI, kg/m^2^ (IQR)	24.8 (22.5–26.2)	
Gender (%)	Female	10 (27.8)
	Male	26 (72.2)
Chief complain (%)	Incidental finding	32 (88.8)
	Abdominal pain	2 (5.6)
	Flank pain	2 (5.6)
Hypertension (%)	22 (61.1)	
Diabetes (%)	8 (22.2)	
End-stage renal disease (%)	7 (19.4)	
CCI (IQR)	1 (0–2)	
ECOG PS 0 (%)	36 (100)	
Smoking (%)	Current smoker	4 (11.1)
	Ex-smoker	14 (38.9)
	Non-smoker	18 (50.0)
VHL syndrome (%)	1 (2.8)	
Synchronous ipsilateral multiple mass (%)	3 (8.3)	
Synchronous bilateral mass (%)	1 (2.8)	
Metachronous CCPRCT (%)	1 (2.8)	
Metachronous ccRCC or other type (%)	4 (11.1)	
Synchronous ccRCC (%)	1 (2.8)	

A total of 38 surgeries were performed because each side was operated separately in 2 patients. Nineteen cases (50%) were performed on the right and left sides respectively. The median maximum diameter of the mass was 1.2 (IQR: 1.0-1.7) cm, and 4 cm or more was found in one case. There was no thrombosis or metastasis of the tumor on preoperative imaging. Partial nephrectomy was performed 30 times in 29 patients (78.9%). Radical nephrectomy was performed 8 times in 7 patients (21.1%), all of whom had end-stage renal disease. Fuhrman grade was measured for each mass. There were 5 masses (12.2%) for Grade 1, 29 masses (70.7%) for Grade 2, and 7 masses (17.1%) for Grade 3, respectively. Characteristics of renal masses are shown in Table 2. In the case of immunohistochemical staining, CD10 was negative in 83.3%, and CK7 was positive in all cases. In AMACR, 13 (92.9%), except one case, were negative and all TFE3 were negative. [Table 2]

**Table 2:** Characteristics of masses

	Per protocols (N = 38)	
Laterality (%)	Left	19 (50)
	Right	19 (50)
Largest diameter of the mass, cm (IQR)	1.2 (1.0-1.7)	
Clinical T stage (%)		
T1a	37 (97.4)	
T1b	1 (1.6)	
Clinical N0, M0 (%)	38 (100)	
Operation Type (%)	Partial nephrectomy	30 (78.9)
	Radical nephrectomy**	8 (21.1)
Fuhrman grade (%)***	Grade 1	5 (12.2)
	Grade 2	29 (70.7)
	Grade 3	7 (17.1)
Immunohistochemical results of CCPRCT		
CD 10, 30 cases (%)	Negative	25 (83.3)
Cytokerain 7, 30 cases (%)	Positive	30 (100)
AMACR, 14 cases (%)	Negative	13 (92.9)
TFE3, 7 cases (%)	Negative	7 (100)

*IQR: Interquatile range; CCPRCT: clear cell papillary renal cell tumor; AMACR: alpha-methylacyl-CoA racemase; TFE3: transcription factor E3

**Performed in patients with end-stage renal disease

***Multiple masses were included

Twenty-nine patients underwent partial nephrectomy. Bilateral masses were partially operated twice. Therefore, the total protocol is 30 times. The median R.E.N.A.L nephrectomy score was 5 (IQR: 4.3-7). Open surgery was performed in 17 cases (56.7%), robot-assisted surgery in 12 cases (40%), and retroperitoneoscopic surgery in 1 case (3.3%), respectively. The median operative time for patients who underwent partial nephrectomy was 97.5 min (IQR: 70–115) and the median ischemic time was 13 min (IQR: 11-17.8). Warm ischemia was used in all cases. The estimated blood loss was 100 cc (IQR: 62.5–150). The median hospital days was 4 (IQR: 4–4) days. Moreover, 30-day complications were 2 cases with clavien-dindo classification III or higher. One patient had a ureteral stent and percutaneous drainage insertion due to urinoma, and the other patient had a percutaneous drainage insertion due to a hematoma near the surgical site. Table 3 shows the surgical outcomes of partial nephrectomy.

**Table 3:** Perioperative outcomes of Partial nephrectomy for CCPRCT

	Per protocol (N = 30)		
R.E.N.A.L score (IQR)	5 (4.3-7)		
Operation modality (%)	Open	17 (56.7)	
	Robot-assisted	12 (40)	
	Retroperitoneoscope	1 (3.3)	
Operation time, min (IQR)	97.5 (70–115)		
Ischemic time, min (IQR)	13 (11-17.8)		
Ischemic type (%)	Warm	30 (100)	
Estimated blood loss (IQR) (cc)	100 (62.5–150)		
Hospital days (IQR)	4 (4–4)		
Transfusion (%)	0 (0)		
Conversion to Radical nephrectomy (%)	0 (0)		
Tumor size (IQR) (cm)	1.2 (1-1.9)		
Pathological T stage (%)			
T1a	29 (96.7)		
T1b	1 (3.3)		
Surgical margin (%)	Negative	29 (96.7)	
	Positive	1 (3.3)	
30 days complication, clavien-dindo classification 3 or more	2 (3.3)		

*IQR: Interquatile range; CCPRCT: clear cell papillary renal cell tumor

During the follow-up period, there was no recurrence and disease progression. Moreover, there was no cancer specific mortality. Two died of other causes, the causes being myocardial infarction and gastric cancer, respectively.

## Discussion

Although there have been several pathological studies of CCPRCT, few studies have focused on the patient’s clinical and surgical outcomes. In this study, most patients were discovered incidentally, but about 10% complained of nonspecific flank pain and abdominal pain. Similar results have been found in other studies [8]. However, the diameter of the largest mass is 4.1 cm, and the median diameter of mass is 1.2 cm. It is difficult to prove a clear causal relationship between pain and masses. Although it is known that there is no difference according to gender, the proportion of men in this study was about 70% [11].

Initially, CCPRCT was thought to occur only in ESRD. However, several studies have reported that CCPRCT also occurs in patients without ESRD [12, 13]. Only about 20% of the patients in this study had ESRD as a comorbidity. CCPRCT may present as multiple bilateral disease, and VHL syndrome may require differential diagnosis. CCPRCT has been described in a patient with VHL syndrome [14]. In this study, one patient had VHL syndrome and had a history of contralateral metachronous ccRCC.

Among cases of nephrectomy, CCPRCT accounted for 1.8%. The prevalence of CCPRCT is known to be about 1–4% [8], and it is judged that similar results could be obtained in this cohort even if patients who only undergo actual biopsy and have not undergone surgery are included. CCPRCT has rarely been reported as a metastatic or advanced disease to date [8, 15–17]. There were no advanced stages in this study, too. Therefore, if CCPRCT can be predicted in advance, not only partial nephrectomy but also minimally invasive treatment such as ablation or active surveillance can be considered as treatment options. However, it is not easy to clearly distinguish between CCPRCT and small renal mass with the other histologic subtype on CT or magnetic resonance imaging [18]. If there is no significant change in the image during the follow-up period, it would be a good strategy to establish a treatment plan after confirming the pathology through a biopsy [19].

CCPRCT is mostly small, with an average size reported of about 2 cm. It is usually surrounded by a well-defined, thin fibrocystic mass, and most cases are solitary tumors. Therefore, in patients with remaining renal function, nephron-sparing surgery, also called partial nephrectomy, should be considered. Sometimes, multiple or bilateral tumors have been reported [20, 21]. Multiple and bilateral masses were also reported in this study. In histopathology, CCPRCT consists of variable architectures of solid, cystic and papillary patterns. In immunohistochemistry, it is positive for carbonic anhydrase IX, CK7, and high molecular weight cytokertin, and negative for AMACR, CD10, and TFE3 [7]. Based on these characteristics, ccRCC, papillary RCC, Xp11 translocation RCC, MiT family translocation-associated RCC, and RCC with smooth muscle stroma should be considered as differential diagnoses [22, 23].

This study is significant in that it summarized and described the clinical and preoperative data of CCPRCT patients from the clinician’s point of view. Being able to have an understanding of and impression of pathological features or clinical outcomes of CCPRCT will help develop beneficial treatment plans for patients. In particular, the strength of this study is that it provided surgical information on these pathological results by describing the results of partial nephrectomy at CCPRCT.

This study has some limitations. First, it is retrospective, single-center data, and it did not include a large number of patients. Therefore, the results of our study must be confirmed and validated with a prospective large-scale multi-center study. However, the cohort used in this study is prospectively collected data and it is judged to have less bias than the original retrospective study. Furthermore, although it was meaningful in characterizing and describing CCPRCT, it could not predict preoperative CCPRCT. There was no significant difference on imaging, and the symptoms complained of by the patient were not clear. It was thought that further study was needed to predict CCPRCT using magnetic resonance imaging or contrast enhanced ultrasonography.

## Conclusions

The size of CCPRCT was small, and a mass larger than 4 cm was found in only one case. There were no advanced stage at that time of diagnosis. There was no recurrence or cancer specific mortality during the follow-up period. However, it was difficult to identify in advance through CT image. Further well designed study, multi-center, prospective, and large scale, is needed in the future.

## Data Availability

The datasets used and/or analysed during the current study available from the corresponding author on reasonable request.

## Abbreviations

CCPRCT: Clear cell papillary renal cell tumor
ccRCC: clear cell renal cell carcinoma
CK7: cytokeratin 7
AMACR: alpha-methylacylCoA racemase
VHL: Von Hippel Lindau
